# Prevalence and Associated Factors of Perinatal Depression in Ethiopia: A Systematic Review and Meta-Analysis

**DOI:** 10.1155/2018/1813834

**Published:** 2018-06-19

**Authors:** Amanual Getnet Mersha, Sileshi Ayele Abebe, Lamessa Melese Sori, Tadesse Melaku Abegaz

**Affiliations:** ^1^Department of Gynecology and Obstetrics, School of Medicine, College of Medicine and Health Sciences, University of Gondar, P.O. Box 196, Gondar, Ethiopia; ^2^Department of Psychiatry, College of Medicine and Health Sciences, University of Gondar, P.O. Box 196, Gondar, Ethiopia; ^3^Department of Clinical Pharmacy, School of Pharmacy, College of Medicine and Health Sciences, University of Gondar, P.O. Box 196, Gondar, Ethiopia

## Abstract

**Background:**

There is no pooled evidence regarding the prevalence and potential associated factors of perinatal depression in Ethiopian community. Hence, the current review aimed to examine the prevalence and associated factors of perinatal depression in Ethiopia.

**Method:**

A computerized systematic literature search was made in MEDLINE, Scopus, PubMed, ScienceDirect, and Google Scholar. Each database was searched from its start date to January 2018. All included articles were published in English, which evaluated prevalence and associated factors of perinatal depression in Ethiopia. Pooled estimations with 95% confidence interval (CI) were calculated with DerSimonian-Laird (DL) random-effects model. Publication bias was evaluated by using inspection of funnel plots and statistical tests.

**Result:**

Eight observational studies with an overall sample size of 4624 mothers were included in the review. The pooled prevalence of perinatal depression from these studies reported that the prevalence of perinatal depression in Ethiopia is 25.8% [95% CI, 24.6%-27.1%]. A pervious history of depression [RR: 3.78 (95% CI, 2.18-6.57),* I*^2^ = 41.6%], poor socioeconomic status [RR: 4.67 (95% CI, 2.89-7.53),* I*^2^ = 0%], not living with spouse [RR: 3.76 (95% CI, 1.96-7.38),* I*^2^ = 36.4%], having obstetric complications in previous and/or this pregnancy [RR: 2.74 (95% CI, 1.48-5.06),* I*^2^ = 67.7%], and having unplanned pregnancy [RR: 2.73 (95% CI, 2.11-3.53),* I*^2^ = 0%] were the major factors associated with perinatal depression.

**Conclusion:**

The pooled prevalence of perinatal depression in Ethiopia is far above most developed as well as developing countries. Hence, to realize the sustainable development goals (SDGs) outlined by united nation, much attention should be given to improve maternal mental health through reduction of identified modifiable factors. Maternal health programs, polices, and activities should incorporate maternal mental health as a core component.

## 1. Background

Depression is characterized by feeling of low self-worth, loss of interest, feelings of regret, restlessness, loss of appetite, feelings of fatigue, and poor concentration. Depression is one of the top contributors of global burden of diseases, which affects around 322 million people worldwide and is the leading reason for suicide. WHO Global Health Estimates of 2015 reported that 788,000 people died due to suicide. For every person who dies of suicide, 20 additional people attempted (but did not die by) suicide. Depressive disorders led to a global total of over 50 million Years Lived with Disability (YLD) in 2015 [1, 2]. The estimated prevalence of depression worldwide is increased by 18.4% from 2005 to 2015 [3]. In Ethiopia, depression is the third leading cause of burden of diseases and is also predicted to become the second leading cause of the global disease burden by the year 2020 [4].

Perinatal depression is a nonpsychotic depressive episode ranging from mild to severe symptoms that occur while a woman is pregnant or during postnatal period [5, 6]. The prevalence of perinatal depression varies across different countries. For instance, the prevalence of antepartum depression ranges from 7 to 15% in high-income countries and from 19 to 25% in low-and middle-income countries [7–9]. The prevalence of postpartum depression is twofold higher among women from low- and middle-income countries (20%) as compared to women from high-income countries (10%) [6, 10]. Perinatal depression prevalence as high as 30-50% is reported in South Africa (Chibanda et al., 2010; Hartley et al., 2011; Rochat, Tomlinson, Barnighausen, Newell, and Stein, 2011; Stewart et al., 2010).

One of the main components of sustainable development goal (SDG) is improving maternal health and the vitality of mental health is stated through the theme “*no health without mental health*." In developing countries, one in three to one in five pregnant and postpartum mothers have mental illness [11]. To realize sustainable development goals, efforts must include procedures to avert and manage the issue of maternal mental health during pregnancy and following birth of a baby [12].

Perinatal depression's even milder symptoms impose a considerable health, social, and economic impact on the woman, her family, and her country at large [13, 14]. Most of the core maternal symptoms of perinatal depression such as sleep disturbance and fatigue are frequently attributed to normal response of motherhood, which lowers the detection of perinatal depression [15]. Due to the associated higher risk of using alcohol and substances in such women, they are at increased risk of having obstetric complications such as preterm labor, preeclampsia, fetal growth restriction, abruption placenta, and associated fetal and maternal complications [15–18]. Women having such problems are usually less likely to seek and get care for themselves as well as their child, which in turn leads to preterm birth, low birth weight, and growth restriction [19–21]. In one study conducted in Ethiopia, the odds of low birth weight were found to be 1.87 times higher in women having antepartum depression with 95% CI between 1.09 and 3.21 [22]. A national survey in Ethiopia conducted in 2013 showed an overall unplanned pregnancy rate of 24%.

Women from a developing country are usually exposed to risk factors for the development of perinatal depression like poor socioeconomic status, unintended pregnancy, and gender-based violence [23]. For instance, in Ethiopia, at least one in five women reported intimate partner violence (IPV) [24] and women having history of intimate partner violence (IPV) are 3 to 5 times likely to develop perinatal depression [25–28].

Despite extensive variations in the prevalence and associated factors of perinatal depression across different communities of Ethiopia, there is no pooled evidence regarding the overall prevalence and potential associated factors of perinatal depression. The objective of the current review is to present an overview on the magnitude and associated factors of perinatal depression in Ethiopia.

## 2. Methods

### 2.1. Data Sources and Search Strategy

Articles included in the review were searched from the following databases: MEDLINE, Scopus, PubMed, ScienceDirect, and Google Scholar. Each database was searched from its start date to January 2018 by using the following words: Depression (Mesh), depress*∗*(all fields), “ante partum depression” (Mesh), “postpartum depression” (Mesh), “postnatal depression” (Mesh), Ethiopia(Mesh), “perinatal depression” (Mesh), “pregnancy and depression” (Mesh), mental health (all fields), mental ill*∗*(all fields). The literature search was conducted by two separate researchers (Amanual Getnet Mersha and Tadesse Melaku Abegaz) to avoid missing of articles.

### 2.2. Study Selection and Eligibility

#### 2.2.1. Study Selection

All duplicated searches were removed using the ENDNOTE software version X5 (Thomson Reuters, USA). Two authors (Amanual Getnet Mersha and Tadesse Melaku Abegaz) screened the titles and abstracts of identified articles by applying the inclusion criteria. Two authors (Amanual Getnet Mersha and Sileshi Ayele Abebe) independently reviewed the full text. Final inclusion of the studies was determined by agreement of both reviewers and when there is disagreement, a third author (Lamessa Melese Sori) was involved. All the authors were involved in the discussion and agreed on the final inclusion.

### 2.3. Inclusion Criteria and Quality Assessment

The PRISMA guidelines protocol was used to write the systematic review [35]. Eligibility criteria were defined as follows: (1) articles available in English, (2) studies conducted in Ethiopia, and (3) perinatal depression included as a primary dependent variable. Studies with small samples size (less than 50 participants) and studies with low quality by using STrengthening the Reporting of OBservational Studies in Epidemiology (STROBE) scale checklist (<75%) were excluded to maintain the quality of the findings [36].

### 2.4. Data Extraction and Statistical Analysis

Data on study design, year of study, and study setting and type of depression were retrieved. Data about prevalence of perinatal depression and possible associated factors were extracted from the eligible articles. Data was extracted by two researches (Amanual Getnet Mersha and Sileshi Ayele Abebe) and cross-checked to minimize error. Data were entered to Comprehensive Meta-Analysis version 2 [37] and analysis was carried out to determine the pooled prevalence of perinatal depression and relative risk of the associated factors by using random-effects model to combine results of included studies in the meta-analysis. The heterogeneity in pooled estimation was determined by using DerSimonian-Laird (DL) method and evaluated using* I*^2^. Sensitivity analysis was also carried out to detect any sources of variation in the pooled estimation. Moreover, publication bias was evaluated by inspection of funnel plots and using Egger and Begg's tests.

## 3. Results

**Figure 1:**
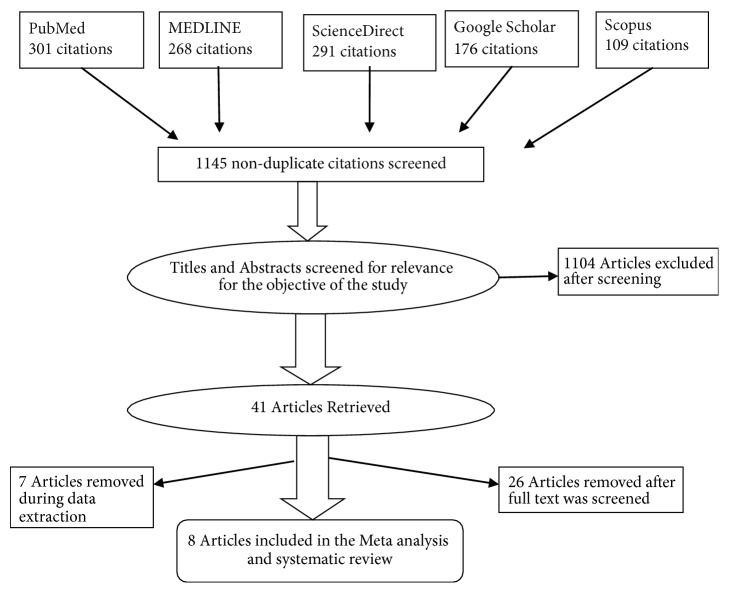
Flowchart indicating the selection process of studies.

A total of 1145 articles were identified from five databases: MEDLINE (268), Scopus (109), PubMed (301), ScienceDirect (291), and Google Scholar (176). After meticulous review of the searched articles, 41 articles were deemed eligible for the full-text review and eight articles were finally included in the systematic review and meta-analysis (Figure 1).

### 3.1. Study Characteristics and Depression Measures

A total of eight articles [29–34, 38, 39] were included in the systematic review and meta-analysis, giving an overall sample size of 4624 mothers. Sample size across the studies ranges from 196 [31] to 1311 mothers [39]. All the studies included in the review were cross-sectional studies. Four of the studies were community-based [30, 32–34] and the other four were facility-based studies [29, 31, 38, 39]. Three of the studies used the Edinburgh Postnatal Depression Scale (EPDS) that considered cutoff point of 13 and above as indicative of depression [29, 33, 34]; two studies used the WHO Self-reporting Questionnaire (SRQ-20) items with a cutoff point of 6 and above to separate cases of perinatal depression. [30, 32]; two studies used Beck Depression Inventory (BDI) as an assessment tool with a score of 16 and more being considered as depressed [31, 38] and only one study used Patient Health Questionnaire (PHQ-9) to assess prevalence of perinatal depression with a score of five or more being indicative of depression [39] (Table 1).

Women's marital statuses were reported to be significantly associated (*p* value < 0.05 at 95% CI) in four of the studies [29–32] and four studies documented plan of pregnancy as a significantly associated factor (*p* value < 0.05 at 95% CI) for the development of perinatal depression [29, 30, 32, 33]. Out of the eight studies, four studies reported that history of depression was significantly associated (*p* value < 0.05 at 95% CI) with perinatal depression [29, 30, 32, 34]. Three studies found that previous/present history of obstetric complications has significant association (*p* value < 0.05 at 95% CI) for the development of perinatal depression [30, 32, 34] and socioeconomic factors were reported to be significantly associated (*p* value < 0.05 at 95% CI) with perinatal depression in three of the studies [31–33] (Table 2).

### 3.2. Prevalence of Perinatal Depression

**Figure 2:**
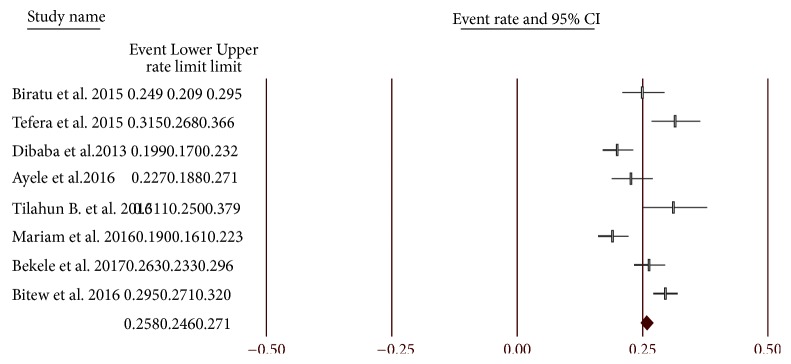
Prevalence of perinatal depression.

The pooled prevalence of perinatal depression from eight studies showed that the prevalence of perinatal depression in Ethiopia is 25.8% [95% CI, 24.6%-27.1%]. The prevalence of perinatal depression ranges between 19% [34] and 31.5% [30] across the studies. Depending on the depression measure used, the pooled prevalence of depression for three studies [29, 33, 34] which used EPDS to diagnose depression was 20.9% (95% CI: 19%-22.9%) and prevalence for two studies [30, 32] which used SRQ-20 to diagnose depression was 28% (95% CI: 25.4%-30.7%). Two studies [31, 38] used BDI as an assessment tool and the pooled prevalence from these two studies was found to be 25.7% (95% CI: 22.3%-29.4%). On the other hand, only one study [39] used PHQ-9 to assess prevalence of perinatal depression, which was shown to be 29.5% (95% CI: 27.1%-32%) (Figure 2).

### 3.3. Associated Factors of Perinatal Depression

As shown in Table 2, having history of depression [RR: 3.78 (95% CI, 2.18-6.57),* I*^2^ = 41.6%], low socioeconomic status [RR: 4.67 (95% CI, 2.89- 7.53),* I*^2^ = 0%], not living with spouse [RR: 3.76 (95% CI, 1.96-7.38),* I*^2^ = 36.4%], having obstetric complications in previous and/or the current pregnancy [RR: 2.74 (95% CI, 1.48-5.06),* I*^2^ = 67.7%], and having unplanned pregnancy [RR: 2.73 (95% CI, 2.11-3.53),* I*^2^ = 0%] were the major factors associated with perinatal depression (Table 2).

### 3.4. Publication Bias and Sensitivity Analysis

**Figure 3:**
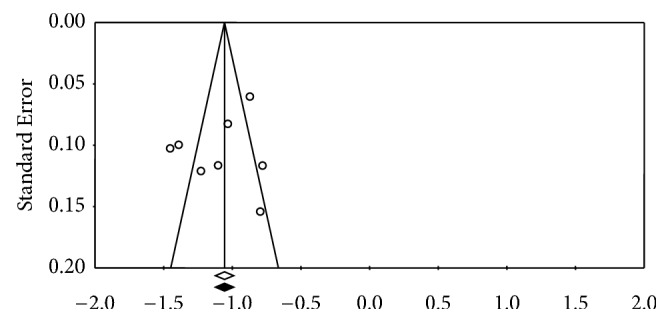
**Funnel plot** (Egger's test *p* value: 0.48; Begg's test: *p*: 0.80).

The sensitivity analysis showed that omission of any of the incorporated studies did not change the pooled results for both prevalence and associated factors (all *p* < 0.05). Funnel plots supplemented by statistical tests confirmed that there existed some evidence of publication bias in the prevalence of perinatal depression (Egger's test, *p* = 0.48; Begg's test, *p* = 0.80) (Figure 3).

## 4. Discussion

The current review is the only review that tried to evaluate the prevalence and associated factors of perinatal depression in Ethiopia. This review demonstrated the high prevalence of perinatal depression among Ethiopian women and it is significantly associated with pervious history of depression, low socioeconomic status, not living with spouse, having obstetric complications in previous and/or this pregnancy, and having unplanned pregnancy.

More than one among four women suffers from perinatal depression in Ethiopia [25.8% (95% CI, 24.6%-27.1%), I^2^-28%]. The pooled prevalence of depression is highest in those studies that used SRQ-20 [28% (95% CI: 25.4%-30.7%)] and PHQ-9 [29.5% (95% CI: 27.1%-32%)] as a screening tool for perinatal depression. This prevalence is comparable with other reviews done in different low- and middle-income countries, 19 to 25% [7–9]. This could be due to the shared lower economic status, higher rate of unplanned pregnancies, and political instability in such setups. This figure is more than two times higher than results reported from high-income countries (10%) [6, 10]. This discrepancy may result from the fact that women in Ethiopia have a poor economic status, higher rate of obstetric complications, higher rate of unplanned pregnancies, and the higher rate of intimate partner violence (IPV) [24]. On the other hand, Parsons and colleagues reported the perinatal depression in Ethiopia to be 13.7% in 2011 global report. First, it incorporated only two studies from Ethiopia. Both of the included studies were conducted in urban setups, which may not reflect the whole picture of the country, especially in countries like Ethiopia, where more than 85% of the population resides in rural areas. It was from a figure taken before about 10 years and 8 years back; hence, it may not reflect the current rate.

The pooled relative risk of having perinatal depression in those women who have a history of depression is found to be 3.78 [(95% CI: 2.18-6.57),* I*^2^ = 41.6%]. Similar results were found from a systematic review done in 2012 among low- and middle-income countries [6, 40]. This can be explained by the fact that most risk factors for the development of depression like poor socioeconomic status are recurrent.

This review illustrated that poor socioeconomic status was a considerably associated factor of perinatal depression and the pooled relative risk of perinatal depression from these studies was found to be 4.67 [(95 CI: 2.89-7.53),* I*^2^ = 0%] in those with poor socioeconomic status. The finding is in agreement with studies conducted in Nepal and Turkey [41, 42]. The association could be due to the fact that food insecurity is a major problem in Ethiopia, where majority of the population is under poverty line [43] and the vast majority of pregnancies are unplanned; it will impose a considerable mental effect on the women.

The review also showed a substantially elevated risk of developing perinatal depression in those women who are not living with their spouse (single, widowed, or separated) with a pooled relative risk of 3.76 [(95 CI: 1.96-7.38),* I*^2^ = 36.4%]. The result is similar to a study done in South Africa [44]. This may be due to absence of economical, physical, and psychological support in those women who live alone.

Women who have history of obstetric complications in previous and/or this pregnancy have a pooled threefold estimated risk of having perinatal depression in comparison to those who did not report such history [2.74 (1.48-5.06),* I*^2^ = 67.7%]. This result is similar to studies conducted in other African countries [45], Brazil [46], and Pakistan [47] and also studies conducted in high-income countries [48]. This can be explained by the women's fear of having the same complications in the current as well as future pregnancies and child birth.

Near to threefold higher pooled estimate of perinatal depression was reported among women who have unplanned pregnancy as compared to those who planned their pregnancy [2.73 (2.11-3.53),* I*^2^ = 0%]. This factor is also reported to be associated with perinatal depression from study done in 2013 in India [49] and a systematic review done in 2012 among low- and middle-income countries [6]. Pregnancy brings a lot of physical, psychological, and physiological changes that need a lot of preparations. Hence, women with unplanned pregnancy are at a higher risk of depression. Beside these changes, economical preparation is also mandatory, especially in women from a low-income country like Ethiopia.

There are also other factors that are reported to have significant association with perinatal depression among Ethiopian women. These factors are being housewife [31, 38], lack of social support [29, 30, 33], women's age group between 20 and 29 years [38], husband being smoker [30], and having history of intimate partner violence during pregnancy [33].

## 5. Limitation of the Study

There is some evidence of heterogeneity and publication bias in the review. It could be due to differences in the study setup; that means half of the studies were health-facility-based and the other half were community-based. There is also a difference in the depression assessment tools, which may also have contribution to the detected heterogeneity in this review. Hence, caution should be taken during interpretation of the results.

## 6. Conclusion and Recommendations

This review demonstrated the high prevalence of perinatal depression among Ethiopian women and it is significantly associated with pervious history of depression, low socioeconomic status, not living with spouse, having obstetric complications in previous and/or this pregnancy, and having unplanned pregnancy. Hence, to realize the sustainable development goal (SDG-5) outlined by United Nations (UN), much attention should be given to improving maternal mental health through reduction of identified modifiable factors. Maternal health programs, polices, and activities should incorporate maternal mental health as a core component. Further researches are recommended to assess other possible associated factors for the high prevalence of perinatal depression in Ethiopia.

## Figures and Tables

**Table 1:** Overview of studies included in the systematic review and meta-analysis.

Source	Year	Location	Type of study	Depression measure	Sample size	Prevalence of MD	Not living with her spouse	Unwanted/unplanned pregnancy	History of depression	Perceived/having complication	Low economic status	% STROBE criteria met
Biratu et al.	2015	Addis Ababa	Cross-sectional	EPDS	393	24.94%	1.799 (0.728-4.441)	2.779 (1.594-4.846)	2.569 (1.475-4.475)	-	-	88%
Tefera et al.	2015	Bale	Cross-sectional	SRQ-20	340	31.5%	4.95 (2.58-9.48)	2.36 (1.47-3.81)	0.27 (0.10-0.72)	0.43 (0.20-0.93)	-	81%
Dibaba et al.	2013	Gilgel Gibe	Cross-sectional	EPDS	627	19.9%	-	1.96 (1.04-3.69).	-	-	4.60 (2.75-7.70)	79%
Ayele et al.	2016	Gondar	Cross-sectional	BDI	388	23%	-	-	-	-	-	81%
Tilahun B. et al.	2016	Maichew	Cross-sectional	BDI	196	31.1%	4.07 (1.18-14.04	-	-	-	5.12 (1.42-18.48)	77%
Mariam et al.	2016	Adigrat	Cross-sectional	EPDS	616	19%	-	-	5.94 (2.944, 11.963)	3.689 (2.351-5.790)	-	76%
Bekele et al.	2017	Saint Paul	Cross-sectional	SRQ-20	753	26.2%	19.64 (1.35-285.07)	3.64 (2.32-5.70)	4.04 (1.44-11.32)	1.97 (1.16-3.37)	0.59 (0.36-0.97)	91%
Bitew et al.	2016	Gurage	Cross-sectional	PHQ-9	1311	29.5%	-	-	-	-	-	76%

BDI: Beck Depression Inventory, PHQ-9: Patient Health Questionnaire, SRQ-20: Self-reported Questionnaire, EPDS: Edinburgh Postnatal Depression Scale.

**Table 2:** Associated factors of perinatal depression in Ethiopia.

Associated factors	Vulnerable group	Effect size (RR and CI)	*I* ^2^	study
Marital status	Single, widowed, separated	3.76 (1.96-7.38)	36.4%	[29–32]
Plan of pregnancy	Unplanned pregnancy	2.73(2.11-3.53)	0%	[29, 30, 32, 33]
History of depression	Having previous history of depression	3.78 (2.18-6.57)	41.6%	[29, 30, 32, 34]
Obstetric complications	Having complications in previous and/or this pregnancy	2.74 (1.48-5.06)	67.7%	[30, 32, 34]
Socioeconomic status	Poor socioeconomic status	4.67 (2.89-7.53)	0%	[31–33]

For all the results, *p* value is < 0.05 at 95% CI (confidence interval).

## Data Availability

All relevant materials and data supporting the findings of this review are contained within the manuscript.

## Abbreviations

CI: Confidence interval
SDG: Sustainable development goal
WHO: World Health Organization
YLD: Years Lived with Disability.
